# A comparison of different machine-learning techniques for the selection of a panel of metabolites allowing early detection of brain tumors

**DOI:** 10.1038/s41598-023-38243-1

**Published:** 2023-07-08

**Authors:** Adrian Godlewski, Marcin Czajkowski, Patrycja Mojsak, Tomasz Pienkowski, Wioleta Gosk, Tomasz Lyson, Zenon Mariak, Joanna Reszec, Marcin Kondraciuk, Karol Kaminski, Marek Kretowski, Marcin Moniuszko, Adam Kretowski, Michal Ciborowski

**Affiliations:** 1 Clinical Research Centre, Medical University of Bialystok, M. Sklodowskiej-Curie 24a, 15-276 Białystok, Poland; 2 Faculty of Computer Science, Bialystok University of Technology, Białystok, Poland; 3 Department of Neurosurgery, Medical University of Bialystok, Białystok, Poland; 4 Department of Medical Pathomorphology, Medical University of Bialystok, Białystok, Poland; 5 Department of Population Medicine and Lifestyle Diseases Prevention, Medical University of Bialystok, Białystok, Poland; 6 Department of Regenerative Medicine and Immune Regulation, Medical University of Bialystok, Białystok, Poland; 7 Department of Allergology and Internal Medicine, Medical University of Bialystok, Białystok, Poland; 8 Department of Endocrinology, Diabetology and Internal Medicine, Medical University of Bialystok, Białystok, Poland

**Keywords:** Biochemistry, Cancer, Computational biology and bioinformatics, Biomarkers, Oncology

## Abstract

Metabolomics combined with machine learning methods (MLMs), is a powerful tool for searching novel diagnostic panels. This study was intended to use targeted plasma metabolomics and advanced MLMs to develop strategies for diagnosing brain tumors. Measurement of 188 metabolites was performed on plasma samples collected from 95 patients with gliomas (grade I–IV), 70 with meningioma, and 71 healthy individuals as a control group. Four predictive models to diagnose glioma were prepared using 10 MLMs and a conventional approach. Based on the cross-validation results of the created models, the F1-scores were calculated, then obtained values were compared. Subsequently, the best algorithm was applied to perform five comparisons involving gliomas, meningiomas, and controls. The best results were obtained using the newly developed hybrid evolutionary heterogeneous decision tree (EvoHDTree) algorithm, which was validated using Leave-One-Out Cross-Validation, resulting in an F1-score for all comparisons in the range of 0.476–0.948 and the area under the ROC curves ranging from 0.660 to 0.873. Brain tumor diagnostic panels were constructed with unique metabolites, which reduces the likelihood of misdiagnosis. This study proposes a novel interdisciplinary method for brain tumor diagnosis based on metabolomics and EvoHDTree, exhibiting significant predictive coefficients.

## Introduction

Gliomas are one of the most common and debilitating primary tumors of the central nervous system (CNS) and are characterized by high mortality and recurrence rate^1,2^. The survival rate depends, among others, on the tumor size and localization, age of manifestation, and molecular genetic factors^3^. According to the 2021 WHO Classification of Tumors of the Central Nervous System, gliomas are classified by their molecular features and can be divided into four grades based on their malignancy^4^. Thus, CNS WHO grade I and II gliomas are commonly considered low-grade gliomas (LGG), while CNS WHO grade III and IV gliomas are considered high-grade gliomas (HGG). Moreover, within 5–10 years from diagnosis, less aggressive at the time of diagnosis LGG, may eventually transform into HGG, increasing the fatality risk^5^. Despite new surgical and therapeutic techniques, the medium survival rate of patients diagnosed with CNS WHO IV glioma ranges from 12 to 15 months^5,6^. Current pre-surgical diagnostic methods may not be sensitive or specific enough to solely depict brain tumor type or grade, contributing to poor prognosis of patients with glioma.

However, brain tumor research should not end on malignant entities solely. The most frequently diagnosed type of primary brain tumor is meningioma (meningeal tumor, MT)^7^. On a completely different origin than glioma, MTs arise from the meninges, a membrane that surrounds the brain and spinal cord. Thus, technically it does not originate from brain cells, but due to its presence may compress or squeeze the adjacent nerves and vessels, it is included in the category of brain tumors^8^. In comparison to gliomas, MTs present a high 5-year survival rate, which is in the range from 87 to 97%, depending on people’s age^9^. However, the statement that MT cannot be malignant is false; some benign or atypical MTs can have clinically aggressive behaviors^4^.

Brain tumor cells, as well as other neoplastic diseases, are based on hallmarks of cancer development proposed by Hanahan and Weinberg^10^. Mainly these are: the limitless ability to proliferate, escape from cell death through altered apoptotic responses, capacity to stimulate angiogenesis, self-sufficiency in growth stimulating signals, insensitivity to classic growth inhibitory signaling, and migratory and invasive behavior^10^. Additionally: genome instability and tumor-promoting inflammation, which both affect cellular energetics and immune evasion^10^. Each of the hallmarks eventually leaves its mark on the metabolome, as the metabolites are the final products of every biochemical pathway. In accordance with the Warburg effect, obtaining nutrients for growth through increased intake of lipids, amino acids (AA), and glucose are leading changes in metabolism affecting global metabolome composition^11^. Consequently, metabolomics could provide information about tumorigenesis and tumor progression by recognizing modified metabolic pathways and altered lipid profiles^2^. Moreover, metabolomics tools are already powerful enough to indicate metabolites that may be useful as a support for glioma diagnosis or distinguishing histological grades and types^12–14^. In the case of MTs, there is scarce research in metabolomics. However, these few indicate that MT metabolomic signatures can be associated with poor prognosis histological markers, such as Ki-67 or progesterone receptor expression^15^. Quantifying in plasma or serum small molecules characteristic to brain tumors would allow for a quicker phenotype determination of cancer in patients, reducing the diagnosis process and hastening the implementation of appropriate treatment^16^. Methods based on mass spectrometry allow for simultaneous monitoring of the concentration of many metabolites, which can give a basis for the development of a highly sensitive and specific diagnostic test^17^. However, primarily, the selection of an appropriate panel of metabolites characteristic of the relevant neoplastic disease is necessary.

Recently, metabolomics techniques are combined with machine learning methods (MLMs), which are used for the preparation of diagnostic or prognostic panels of biomarkers. This trend is an important alternative to the standard statistical methods such as partial least squares regression, which can only be applied to model linear latent covariance^18^. However, biological data are usually non-linear^19^, requiring complex computational algorithms. Non-linear machine learning methods such as random forest (RF) and kernel support vector machine (SVM) may be better suited for extensive amounts of metabolomic data^12,20^. MLMs have already been applied to develop diagnostic methods for such cancers as lung^12^, breast^20^, endometrial^21^, kidney^22^, oral^23^, liver^24^, and prostate^17^. By use of such advanced data analysis techniques it is possible to obtain diagnostic panels with high model effectiveness coefficients. However, the indicated model should be appropriately optimized since its overfitting may lead to falsified results and erroneous conclusions^18^. Moreover, most MLMs tend to focus almost exclusively on prediction accuracy (ACC) and propose complex predictive models. Such an approach hinders the process of uncovering new biological understanding and is often an obstacle for mature applications^25,26^. In this research, we focus on both complex and simple MLMs: Naive Bayes (NB), Generalized Linear Model (GLM), Logistic Regression (LR), Fast Large Margin (FLM), Deep Learning (DL), Decision Tree (DT), RF, Gradient Boosted Trees (GBT), SVM, and Evolutionary Heterogeneous Decision Tree (EvoHDTree)^25^. So far most of them have not been used to indicate gliomas diagnostic panels composed of small molecules. Consequently, the main goal of this study was to develop a glioma diagnostic strategy, notably in LGG, using targeted analysis of metabolites by liquid chromatography coupled with tandem mass spectrometry (LC–MS/MS) in combination with MLMs. We focused on elaborating diagnostic panels that allow the diagnosis of the glioma grades and distinguish a malignant tumor from a non-malignant tumor, such as MT. To our knowledge, this is the first study in which ten MLMs and univariate statistics (UVS) were applied to plasma metabolomics data in order to indicate the best panel of markers for glioma diagnosis.

## Results

The aim of this study was to identify a panel of metabolites that can be used for a routine diagnosis of brain tumors. In the first part of this study, we applied a conventional statistical approach (UVS followed by ROC analysis) and ten MLMs, including the novel EvoHDTree hybrid algorithm, to analyze obtained metabolomics data. We performed four comparisons: patients with grade I and II glioma (GI–II) vs. Con, patients with grade III glioma (GIII) vs. Con, patients with grade IV glioma (GIV) vs. Con, and glioma patients without grade division (GI–IV) vs. Con. The confusion matrices obtained for all comparisons were used to calculate for each method the qualifier evaluation parameter. Subsequently, the obtained values of AUC, ACC, and F1–score were compared to choose the best predictive method. The benchmark of the methods used is shown in Table 1. The F1-score was used to compare applied MLMs since this factor combines the precision and classifier recall into a single metric.

**Table 1:** Comparison of the parameters of ROC curves generated with different MLMs.

	GI-II vs. Con			GIII vs. Con			GIV vs. Con			GI-IV vs. Con		
	AUC	ACC	F1-score	AUC	ACC	F1-score	AUC	ACC	F1-score	AUC	ACC	F1-score
Naive Bayes	0.310	0.820	0.000	0.838	0.693	0.000	0.975	0.925	0.927	0.935	0.849	0.867
Generalized Linear Model	0.925	0.820	0.000	0.863	0.833	0.667	0.988	0.925	0.927	0.910	0.769	0.744
Logistic Regression	0.440	0.820	0.000	0.750	0.833	0.667	0.260	0.314	0.310	0.727	0.673	0.779
Fast Large Margin	0.200	0.820	0.000	0.775	0.653	0.567	1.000	0.921	0.927	0.933	0.853	0.886
Deep Learning	0.590	0.833	0.000	0.800	0.833	0.667	0.988	0.868	0.914	0.930	0.853	0.843
Decision Tree	0.425	0.820	0.000	0.500	0.693	0.000	0.992	0.918	0.909	0.770	0.647	0.768
Random Forest	0.835	0.520	0.454	0.775	0.833	0.667	1.000	0.950	0.956	0.923	0.871	0.866
Gradient Boosted Trees	0.595	0.727	0.000	0.800	0.673	0.000	0.988	0.950	0.950	0.967	0.858	0.871
Support Vector Machine	0.710	0.820	0.000	0.775	0.833	0.667	0.913	0.514	0.667	0.883	0.604	0.747
Conventional approach	0.878	0.787	0.578	0.987	0.975	0.909	0.980	0.942	0.938	0.966	0.933	0.940
EvoHDTree	0.898	0.910	0.714	0.812	0.975	0.889	0.971	0.985	0.985	0.951	0.952	0.958

ACC, accuracy; AUC, area under the curve; Con, healthy control; EvoHDTree, Evolutionary Heterogeneous Decision Tree; GI-IV, I–IV grade glioma.

**Figure 1:**
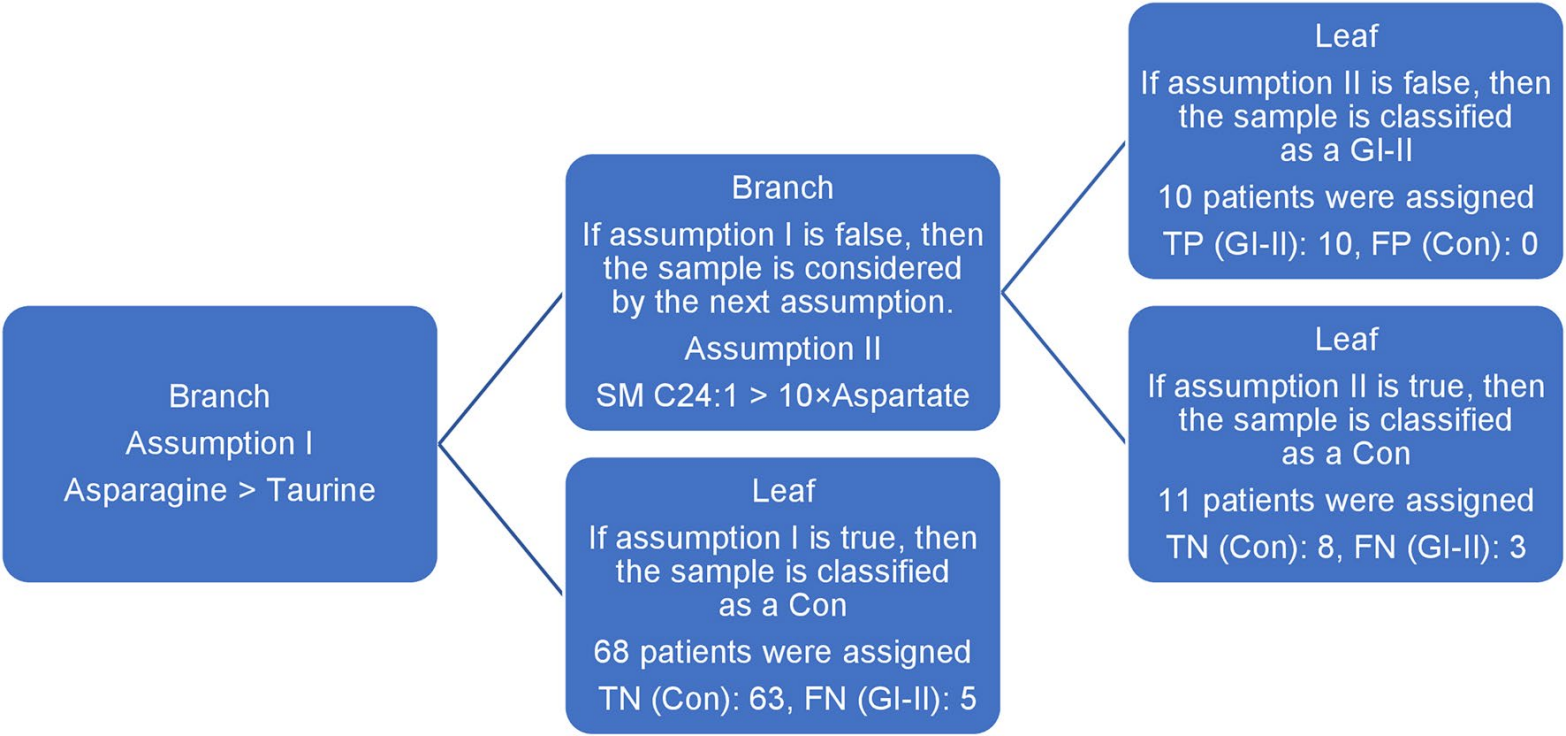
Graphical representation of the principle of the EvoHDTree algorithm using the GI-II vs. Con comparison as an example.

Based on the results presented in Table 1, we can conclude that the highest F1-score for the four comparisons was obtained for the EvoHDTree algorithm (0.714–0.985) and RF (0.454–0.956), respectively. The conventional approach (UVS followed by ROC analysis with SVM) yielded comparable results (F1-score range of 0.578–0.940) to the newly developed hybrid method. The least useful model was created with logistic regression (F1-score range of 0–0.779). ROC analysis based on statistically significant metabolites and EvoHDTree proved to be valuable tools for preparing prediction models. However, considering the results of the GI–II vs. Con comparison, EvoHDTree performed better than the conventional approach. For the EvoHDTree method, the F1-score and ACC values were 0.714 and 0.910, respectively, while for the conventional approach 0.578 and 0.787, respectively. Analysis of the Friedman test results showed statistically significant differences between the algorithms (significance level equal to 0.05) in terms of ACC. According to Dunn’s multiple comparison test, EvoHDTree managed to significantly outperform the other solutions in almost all comparisons. Additionally, as seen in the example of the GI–II vs. Con comparison (Fig. 1), EvoHDTree is an easy-to-understand algorithm, and the obtained results are straightforward to interpret. For this reason, we have chosen the newly developed hybrid algorithm for further analysis.

**Figure 2:**
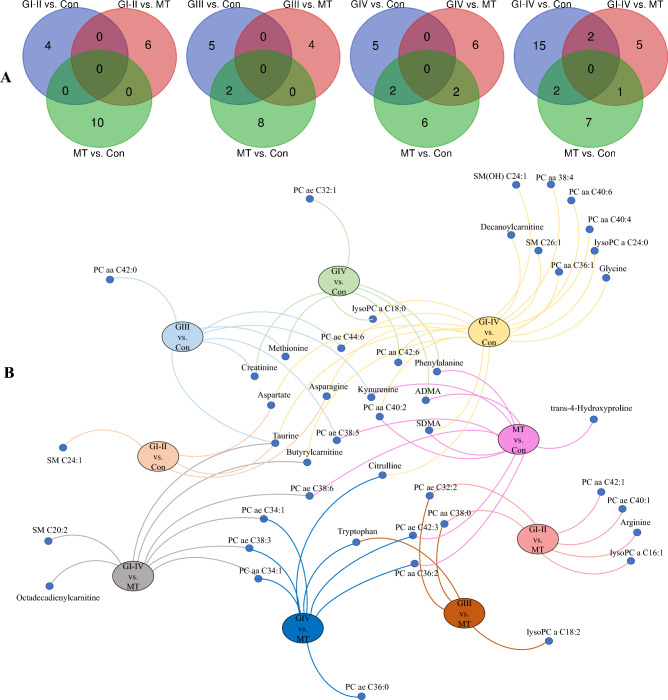
Representation of the extracted metabolites (n = 45) from the EvoHDTree models for all comparisons. (**A**) Venn diagrams indicate the selectivity of metabolites for each comparison. (**B**) Bipartite graphs show small nodes which represent metabolites while large nodes performed comparisons. The connection between the large and small knots means that a given metabolite is included in this comparison. ADMA, asymmetric dimethylarginine; Con, healthy control; GI-IV, I-IV grade glioma; MT, meningioma; PC, phosphatidylcholine; SDMA, symmetric dimethylarginine; SM sphingomyelin.

In the second part of the experiment, we used EvoHDTree to prepare predictive models for the following comparisons: GI–II vs. MT, GIII vs. MT, GIV vs. MT, and Con vs. MT. Obtained models were validated using the cross-validation method and re-validated with the restrictive Leave-One-Out Cross-Validation (LOOCV). A summary of these two validations results is shown in Table 2. As can be seen, obtained values for ACC and F1-score parameters are usually lower when LOOCV validation was performed. It is related to the specificity of this method, which tests each observation separately, not only the test group, as in the case of cross-validation^27^. Metabolites used by EvoHDTree to develop predictive panels for nine comparisons are presented in Fig. 2. Venn diagrams demonstrate the number of selected metabolites by the EvoHDTree algorithm that are considered in the nine comparisons (Fig. 2A). Metabolites composing diagnostic panels for GI-II vs. Con, GI–II vs. MT, and MT vs. Con comparisons were not overlapping.

**Table 2:** Comparison of two types of validation for the EvoHDTree algorithm.

Type of validation	Parameters	GI-II vs. Con	GIII vs. Con	GIV vs. Con	GI-IV vs. Con	GI-II vs. MT	GIII vs. MT	GIV vs. MT	GI-IV vs. MT	MT vs. Con
LOOCV	ACC (SD)	0.888 (0.070)	0.975 (0.040)	0.949 (0.040)	0.873 (0.050)	0.750 (0.090)	0.838 (0.060)	0.779 (0.100)	0.823 (0.080)	0.865 (0.050)
	F1-score	0.667 (0.080)	0.889 (0.040)	0.948 (0.030)	0.889 (0.050)	0.476 (0.100)	0.552 (0.080)	0.815 (0.090)	0.848 (0.070)	0.853 (0.060)
Cross-validation	ACC	0.910 (0.090)	0.975 (0.050)	0.985 (0.040)	0.952 (0.060)	0.966 (0.060)	0.938 (0.080)	0.890 (0.090)	0.860 (0.080)	0.943 (0.050)
	F1-score (SD)	0.714 (0.080)	0.889 (0.050)	0.985 (0.050)	0.958 (0.060)	0.914 (0.070)	0.737 (0.080)	0.878 (0.070)	0.883 (0.070)	0.943 (0.060)

ACC, accuracy; Con, healthy control; GI-IV, I-IV grade glioma; LOOCV, Leave-One-Out Cross-Validation; MT, meningioma; SD, standard deviation.

**Figure 3:**
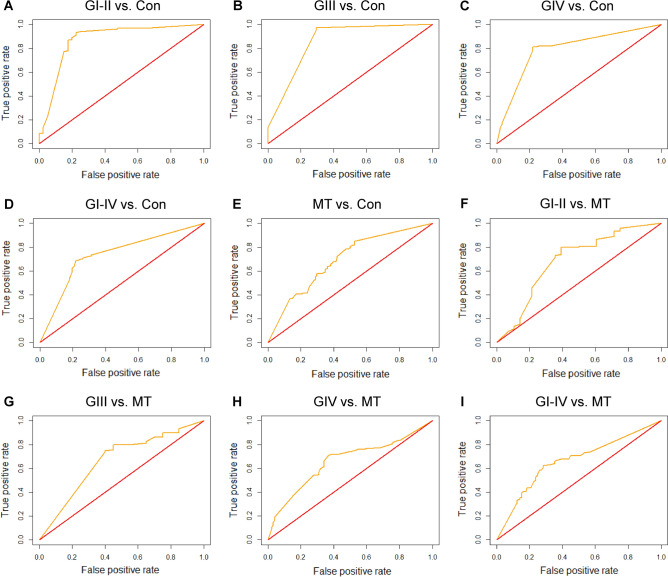
ROC curves for nine comparisons prepared using EvoHDTree. This resulted in: (**A**) AUC = 0.873; (**B**) AUC = 0.857; (**C**) AUC = 0.793; (**D**) AUC = 0.734; (**E**) AUC = 0.694; (**F**) AUC = 0.688; (**G**) AUC = 0.667; (**H**) AUC = 0.660; (**I**) AUC = 0.662. AUC, area under the curve; Con, healthy control; GI-IV, I–IV grade glioma; MT, meningioma.

**Figure 4:**
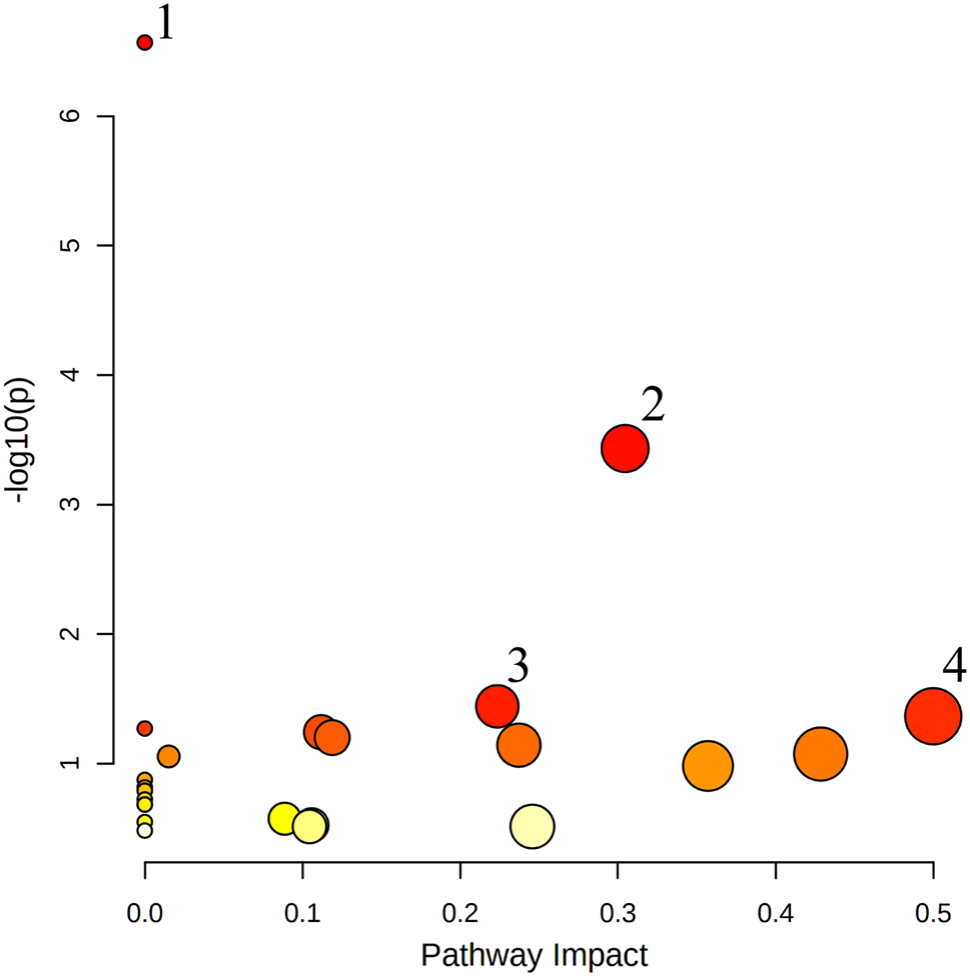
The result from glioma pathway analysis. The analysis showed changes in several metabolic pathways: (1) aminoacyl-tRNA biosynthesis, (2) arginine biosynthesis, (3) alanine, aspartate and glutamate metabolism, (4) phenylalanine, tyrosine and tryptophan biosynthesis.

Finally, using the R programming language, we constructed ROC curves (Fig. 3) for the nine comparisons prepared by EvoHDTree. Summarizing the data collected in Table 2 and shown in Fig. 3 despite the application of LOOCV, the results presented are still characterized by high prediction coefficients. ACC for the nine comparisons ranged from 0.750 to 0.975, and AUC fluctuated from 0.660 to 0.873. In addition, in order to confirm the correct selection of metabolites by EvoHDTree, we performed biochemical pathway analysis using the online tool MetaboAnalyst 5.0. For pathway analysis, we included 45 metabolites (Fig. 2B) extracted from the newly developed hybrid algorithm. We observed changes mainly in four biological pathways (Table S1). These are aminoacyl-tRNA biosynthesis, arginine biosynthesis, alanine, aspartate and glutamate metabolism, and phenylalanine, tyrosine and tryptophan biosynthesis. The overview of pathway analysis is shown in Fig. 4.

## Discussion

Malignant gliomas are responsible for the majority of deaths associated with primary brain tumors. However, early diagnosis could improve the survival rate^28^. In recent years, significant progress has been made in understanding the fundamental metabolic changes related to glioma progression and biology^2,29^. Still, a reliable and accurate method for preoperative brain tumor identification has yet to be developed. Based on the literature review, it was confirmed that analysis of changes in blood metabolite profiles could be an attractive approach to discovering valuable novel glioma biomarkers^2,30^. It has been proven that targeted metabolomics analysis based on mass spectrometry may become a useful diagnostic platform in clinical practices due to its high sensitivity and effective throughput^31^. Therefore, aiming to improve brain tumors diagnosis, we used a targeted metabolomics approach (AbsoluteIDQ p180 kit), which allows quantification of up to 188 metabolites from 6 compound classes (AAs, biogenic amines, acylcarnitines, lysophosphatidylcholines, phosphatidylcholines (PC), sphingolipids, and sum of hexoses) for metabolic profiling of plasma samples of people with glioma, MT, and Con. However, working with biomedical data generated by high-throughput technology, such as the one used in this study, can be challenging due to its large size as well as enormous dimensionality, and natural diversity^26,32^. In this work, MLM was applied to consider all the presented variables during a brain tumor diagnostic strategy development.

Machine learning approaches are becoming of interest to provide actionable knowledge from large data sets generated using LC–MS/MS methods and to improve metabolic profiling endeavors. To the best of our knowledge, this study is the first to compare 10 different supervised MLMs, including the newly developed hybrid method (EvoHDTree), with the conventional approach to determine metabolomics-based prognostic signatures in gliomas. Previously, conventional approaches were widely used in the metabolomics studies of various diseases^13,33^. Currently, novel machine learning algorithms are gaining popularity for constructing predictive methods for various types of cancer^12,17,20–24,34–40^.

Decision trees are one of the most popular “white box” prediction techniques^41^. The success of tree-based approaches can be explained by their effectiveness, ease of interpretation, and extraction of possible diagnostic rules. However, according to recent literature reports, they could not be compatible with current biological data generated by high-throughput technologies due to the enormous dimensionality, experimental noise, and other perturbations^25,32^. For this reason, we proposed a new solution, EvoHDTree, combining DT techniques with evolutionary algorithms and the recently developed concept—RXA. This approach performed very well in the case of genomics data^25^. Therefore, it seemed reasonable to use it to analyze other omics data, namely metabolomics data. This innovative approach made it possible to prepare glioma diagnostic panels with high predictive coefficients.

Comparing the results (Table 1) for the four comparisons (different glioma grades vs. Con) for all the algorithms applied, we concluded that similar results were obtained using EvoHDTree and the conventional approach. Diagnosing a patient with LGG increases the likelihood of a cure before it transforms into HGG and thus significantly increases the chances of survival^5^. For this reason, we focused on the GI–II vs. Con comparison, in which we obtained better results using the new hybrid algorithm. Although the other machine learning methods utilized in this study identified a variety of discriminating metabolites, these methods yielded a considerably larger number of metabolites composing the diagnostic panels, which can make interpretation and subsequent application more challenging. A larger pool of discriminative features may initially appear beneficial, but it carries the risk of overfitting. In addition, the EvoHDTree algorithm selectively selected metabolites to construct predictive models to avoid repetition in each comparison (Fig. 2A), thus, we applied this method for the second part of the experiment. The unique composition of metabolites chosen for each comparison increases the possibility of distinguishing gliomas from MT. Notably, its novelty consists in its flexible tree node representation, which involves both classical univariate and bivariate tests inspired by the RXA concept. Furthermore, we improved evolutionary exploration and exploitation by incorporating our knowledge of decision tree induction and RXA methodology and designing more than a dozen specialized variants of recombination operators.

In the second part of the experiment, we used EvoHDTree to perform four comparisons between gliomas and MTs, as well as MT vs. Con. The purpose of this section was to assess whether there is an overlap between the metabolites used to construct the diagnostic panels for glioma and MT. Applying the same metabolites to distinguish brain tumors could introduce a bias and lead to misdiagnosis. Considering this, we have developed panels of metabolites that can distinguish glioma patients from MT subjects. Subsequently, we again validated nine predictive models using the LOOCV method to verify the obtained results. Despite the restrictive validation method employed, the ACC results obtained for the nine comparisons are still characterized by high predictive coefficients falling within the range of 0.750–0.975. LOOCV is widely regarded as an excellent tool to validate MLM properly in studies based on smaller study groups^42^. Niu et al.^43^ reported that there is no need to divide the dataset into a training set and a test set if the quality of the model is tested using the jackknife test (LOOCV), since the result obtained is a combination of many different independent tests of the dataset. Therefore, LOOCV is increasingly recognized and widely applied by researchers to test the power of prediction methods, despite the drawback of long computation time.

Early glioma detection ensures faster implementation of treatment and thus may contribute to prolonged survival^30^. Therefore, our study focused on a comparison involving LGG and Con. A diagnostic panel for GI-II vs. Con comparison prepared with the use of the EvoHDTree hybrid algorithm mainly used four metabolites (Fig. 1). These were three AAs (taurine, aspartate, asparagine) and sphingomyelin (SM) C24:1. Recently, differences in the levels of certain AAs in the blood of patients with glioma compared to Con have been demonstrated^44,45^. In our study, increased levels of SM C24:1 and asparagine and decreased levels of aspartate and taurine in GI-II vs. Con comparison were observed. According to Jothi et al.^6^, taurine occupied the top-most position in discriminating the grades of gliomas, followed by other AAs such as creatinine and glutamine. In addition, taurine has been considered a potential marker of apoptosis in gliomas^46^. Taurine exhibits antineoplastic and antioxidant properties, but its primary role is osmoregulation^47^. Moreover, taurine is presumed to be a determinant nutritional molecule during the regeneration and development of the central nervous system^48^. The decrease in aspartate with glioma grade growth is due to the conversion of this AA to asparagine using asparagine synthetase. Asparagine, as Thomas et al.^49^ proposed, is a crucial factor in brain tumor growth under nutrient-deprived conditions. In parallel to AA metabolism, our study also highlighted the role of lipids in this disease. In our study, SM C24:1 was positively correlated with tumor aggressiveness due to increasing mean concentration values of this lipid in subsequent glioma vs. Con comparisons. Based on a literature review, further tumor growth after the initiation of tumorigenesis is possible due to the evasion of effector cells, which is enabled through an increase in SM concentration in the cell surface membrane. Partial inhibition of the SM conversion to ceramide, an essential signaling molecule for tumor biology, cell proliferation, apoptosis, aging, and cell migration, facilitates tumor progression^50–52^.

Subsequent comparisons regarding HGG and Con prepared by the EvoHDTree algorithm were based on seven metabolites. For GIII vs. Con, these were kynurenine, creatinine, taurine, methionine, and PCs such as PC ae C44:6, PC aa C42:0, PC ae C38:5. Panels for the GIV vs. Con comparison were built using methionine, creatinine, phenylalanine, asymmetric dimethylarginine (ADMA), PC ae C32:1, PC aa C42:6, lysoPC a C18:0. In our study, upregulation of ADMA, phenylalanine, methionine, and almost all lipids and downregulation of PC aa C42:6, lysoPC a C18:0, kynurenine, and creatinine were observed in comparisons of HGG vs. Con. Du et al.^53^ demonstrated that the Indoleamine 2,3-dioxygenase 1/tryptophan 2,3-dioxygenase signaling pathway accounted for kynurenine release may regulate the expression of aquaporin 4, promoting motility of glioma cells. Additionally, Samanic et al.^54^ reported that in gliomas, the tryptophan/kynurenine ratio was positively correlated with the pathologic grades, which emphasized the perturbation in the kynurenine pathway in gliomas. ADMA, however, is involved in the dimethylarginine dimethylaminohydrolase/ADMA/nitric oxide pathway. Perturbation of this pathway can result in increased local availability of nitric oxide, which promotes tumor angiogenesis, as well as growth, invasion, and metastasis^55^. Moreover, Gorynska et al.^16^ reported the possibility of using solid-phase microextraction during metabolomic phenotyping of gliomas and proved the evidence for disruption of the phenylalanine metabolism pathway. Gorynska et al.^16^ found also that methionine disruption can be correlated with gliomas harboring 1p19q codeletion. Tumor-initiating cells in heterogeneous tumors exhibit increased methionine cycle activity driven by increased methionine adenosyltransferase 2A, which converts methionine to S-adenosylmethionine^56^. Creatine has been shown to be the sole precursor of creatinine. During an irreversible non-enzymatic reaction, creatine is converted to creatinine, which is excreted by the kidneys with the urine^57^. The decrease in creatine was observed in a study by Kinoshita et al.^58^ where they used nuclear magnetic resonance spectroscopy to compare brain tumor sections to normal cortex. Downregulation of creatinine levels in gliomas compared to Con may be associated with malnutrition or muscle atrophy, as it was presented by das Neves et al.^59^ in patients with non-small-cell lung cancer. Li et al.^60^ in their study show that the levels of some PCs (PC aa C38:4, PC aa C 36:3, PC aa C 38:6) and lysoPC a C18:0 in glioma tissue were higher than in control samples. Our study shows that the concentrations of lysoPC a C18:0 in the examined plasma were similar in GI, GII, GIII, MT, and control samples. However, the concentration of this lysoPC significantly decreased in G4 plasma samples, suggesting an increased accumulation of these lipids in HGG. Interestingly, Li et al*.*^60^ found an absence of PC aa C36:1 in glioma tissues compared to control brain tissues. In contrast, Yu et al*.*^61^ proved that PC (36:1) showed lower levels in glioma tissues than in parietal lobe tissues. The literature reports include information on changes in the lipidomic profile of glioma concerning glycerolipids, prenol lipids, cholesterol lipids, phospholipids, and sphingolipids. For this reason, altered lipid metabolism may affect the molecular phenotype of glioma^60^.

A diagnostic panel to distinguish MT from Con was prepared using: kynurenine, symmetric dimethylarginine (SDMA), ADMA, phenylalanine, trans-4-hydroxyproline, and phosphatidylcholines. Concentrations of kynurenine, trans-4-hydroxyproline, PC ae C38:6, PC aa C40:2, and PC aa C36:2 were higher in Con plasma than in MT. In contrast, concentrations of SDMA, ADMA, phenylalanine, PC ae C38:5, and PC ae C42:3 were lower in Con. However, to discriminate glioma from MT using EvoHDTree, we developed four diagnostic panels based mainly on lipid compounds (PCs, lysoPCs, and SMs), four AAs (arginine, tryptophan, taurine, and citrulline), and two acylcarnitines (butyrylcarnitine and octadecadienylcarnitine). Few metabolomics studies on MTs have been published. Gorynska et al*.*^16^, in their study of glioma and MT tissues, reported that patients with MTs had higher levels of aspartic acid, lysine, and arginine. Most metabolomics work on MTs has been done using nuclear magnetic resonance spectroscopy^15,62–64^. Baranovicova et al*.*^63^ used RF to build ROC curves to distinguish MT from Con. They used five metabolites for this purpose: creatine, pyruvate, citrate, formate, and glucose. In their paper, Monleon et al*.*^62^ describe that the metabolic phenotype of MTs with complex karyotypes exhibits standard features of aggressive tumor biochemistry, including increased turnover of membrane metabolites and high glycolytic activity. Decreased levels of ascorbate and glucose and increased lactate levels suggest a greater reliance on anaerobic pyruvate breakdown, indicating a locally hypoxic microenvironment^62^. Moreover, Ijare et al*.*^64^, in their study, indicated that alanine, glutamine, and glutamate were significantly elevated in MT grade II. They also demonstrated that blocking glutamine metabolism with the GLS1 inhibitor led to a decrease in meningioma cell proliferation. Interestingly, the higher glutamine metabolism observed in MT grade 1 resulted in improved sensitivity to treatment^64^.

Additionally, pathway analysis was performed to better understand small molecules dysregulation, which may be a source of potential specific early disturbances, possibly associated with the development of glioma. Through the pathway analysis we identified four the most important altered metabolic pathways, namely: (1) aminoacyl-tRNA biosynthesis, (2) arginine biosynthesis, (3) alanine, aspartate, and glutamate metabolism, (4) phenylalanine, tyrosine, and tryptophan biosynthesis (Fig. 4). These pathways are involved in the regulation of cell proliferation, survival, differentiation, and angiogenesis. The same biochemical pathways were found perturbed in gliomas in other studies^1,16,65−67^.

However, this work has some limitations. The small number of LGG patients may have an impact on the validity of the statistical tests. Another potential limitation is the outdated classification of gliomas. In May 2021, WHO published a new tumor classification of the CNS, based on histological features and genetically defined mutation status^4,68^. In our experiment, patients were recruited before the publication of the novel WHO classification, thus the diagnosis was performed according to the actual classification at that time. Although promising, the obtained results require validation in a larger cohort of patients of different ethnicities and grouped based on the new classification. A larger cohort would allow more variation of cases to be indicated to algorithms at the learning stage.

In conclusion, this study provides a new strategy for LGG diagnosis using targeted plasma analysis based on LC–MS/MS and the newly developed hybrid EvoHDTree method. Thanks to this innovative approach, it was possible to prepare diagnostic panels with high predictive coefficients. In the future, the hybrid algorithm we applied could be adapted to other cancers apart from gliomas.

## Material and methods

### Patients and groups

A total of 240 subjects recruited between 2016 and 2021 from the Department of Neurosurgery of the Medical University of Bialystok were included in the experiment. Patients with incomplete clinical information were rejected. Finally, 94 patients with gliomas (GI = 3, GII = 15, GIII = 10, GIV = 66) and 70 with MT were included. Due to the collection date, the histopathological examination of the surgical sections determined the degree of advancement of individual tumors based on the fourth edition of the WHO Classification of Tumors and the Central Nervous System published in 2016^69^. Then, based on body mass index (BMI), gender, age, and the absence of comorbidities and addictions, 71 subjects undergoing routine health testing within the general population-based cohort study—Bialystok PLUS were entered into the experiment as healthy controls (Con). A total of 235 samples were used to develop diagnostic panels. The characteristics of patients and controls are shown in Table 3. The study was approved by the Ethics Committee of the Medical University of Bialystok (No. APK.002.103.2022). Each participant signed informed consent before sample collection. The investigation conforms with the principles outlined in the Declaration of Helsinki.

**Table 3:** Median clinical parameters of the studied groups.

Parameter	Gliomas	MT	Con	p value*
Total number of patients (male/female)	94 (54/40)	70 (21/49)	71 (34/37)	–
Age [year] (Q1–Q3)	59.00 (49.00–66.00)	60.50 (48.25–68.00)	59.00 (48.00–64.00)	0.4704
BMI [kg/m^2^] (Q1–Q3)	26.83 (23.69–29.73)	26.81 (23.94–29.41)	26.80 (24.40–29.80)	0.7952
WBC [× 10^3^/μL] (Q1–Q3)	10.77 (7.27–14.43)	7.64 (5.92–10.68)	5.70 (4.80–6.55)	< 0.0001
RBC [× 10^6^/μL] (Q1–Q3)	4.73 (4.40–5.02)	4.53 (4.25–4.78)	4.67 (4.37–5.02)	0.0229
HGB [g/dL] (Q1–Q3)	14.25 (13.58–15.30)	13.75 (12.90–14.43)	13.70 (13.20–14.90)	0.0343
HCT [%] (Q1–Q3)	41.40 (38.95–44.00)	40.15 (38.25–42.33)	41.30 (39.05–43.70)	0.1057
MCV [fL] (Q1–Q3)	87.85 (85.80–90.23)	89.05 (86.20–91.90)	88.50 (86.20–91.15)	0.1338
MCH [pg] (Q1–Q3)	30.45 (29.48–31.20)	30.40 (29.50–31.80)	30.10 (28.75–30.90)	0.0914
MCHC [g/dL] (Q1–Q3)	34.30 (33.80–35.10)	34.10 (33.50–34.73)	33.70 (33.00–34.75)	< 0.0001
RDW-SD [fL] (Q1–Q3)	41.70 (40.20–44.23)	42.35 (41.00–45.18)	43.60 (41.60–45.45)	0.0040
RDW-CV [%] (Q1–Q3)	13.00 (12.50–13.60)	12.95 (12.5–13.7)	13.80 (13.40–14.45)	< 0.0001
PLT [× 10^3^/μL] (Q1–Q3)	227.50 (193.80–270.50)	234.50 (191.50–277.50)	226.00 (193.00–266.50)	0.9391
PCT [%] (Q1–Q3)	0.24 (0.21–0.29)	0.25 (0.22–0.29)	0.18 (0.16–0.21)	< 0.0001
MPV [fL] (Q1–Q3)	10.45 (9.70–11.00)	10.60 (10.10–11.30)	8.10 (7.50–8.60)	< 0.0001
PDW [fL] (Q1–Q3)	12.25 (10.78–13.50)	12.45 (11.30–13.80)	14.30 (13.70–15.15)	< 0.0001

BMI, body mass index; Con, healthy control; HCT, hematocrit; HGB, hemoglobin; MCH, mean cell hemoglobin; MCHC, mean corpuscular hemoglobin concentration; MCV, mean corpuscular volume; MPV, mean platelet volume; MT, meningioma; PCT, procalcitonin; PDW, platelet distribution width; PLT, platelet count; PCT, procalcitonin; RBC, red blood cells; RDW-CV, red cell distribution width—coefficient of variation; RDW-SD, red cell distribution width—standard deviation; WBC, white blood cell.

*Kruskal–Wallis non-parametric ANOVA with Dunn's post-correction were performed to obtain presented p-values.

### Targeted metabolomic study using LC–MS/MS

Measurement of 188 plasma metabolites was performed using the AbsoluteIDQ p180 kit from Biocrates Life Sciences AG (Innsbruck, Austria) according to the protocol provided by the manufacturer^70^. Briefly, after dissolving the quality control (QC) samples, the calibration standards, and the mixture of internal standards (ISTD) in water, 10 µL of ISTD was added to each well of the 96-well plate. In the following step, plasma samples, QCs, blank samples, and ISTD were added to the appropriate wells in the extraction plate. After evaporation in a vacuum concentrator (SpeedVac Concentrator, Thermo Fisher Scientific, Savant SPD2010), samples were derivatized with a mixture of ethanol, water, pyridine, and phenyl isothiocyanate. Following the evaporation of the reaction mixture, the analytes from the filters were extracted with 5 mM ammonium acetate in methanol. For analysis by LC–MS/MS, 150 µL of the extract was diluted with the same volume of water. However, in the case of flow-injection analysis coupled with tandem mass spectrometry, the extract was diluted in a 1:49 ratio with the solvent supplied with the kit.

Samples were analyzed in a randomized order in three batches using ultrahigh performance liquid chromatography (1290 Infinity II, Agilent Technology, Santa Clara, CA, USA) coupled with a tandem mass spectrometer (6470 Triple Quad LC/MS, Agilent Technologies, Santa Clara, CA, USA). LC–MS/MS was operated in positive polarity in multiple reaction monitoring mode.

### Data treatment

**Figure 5:**
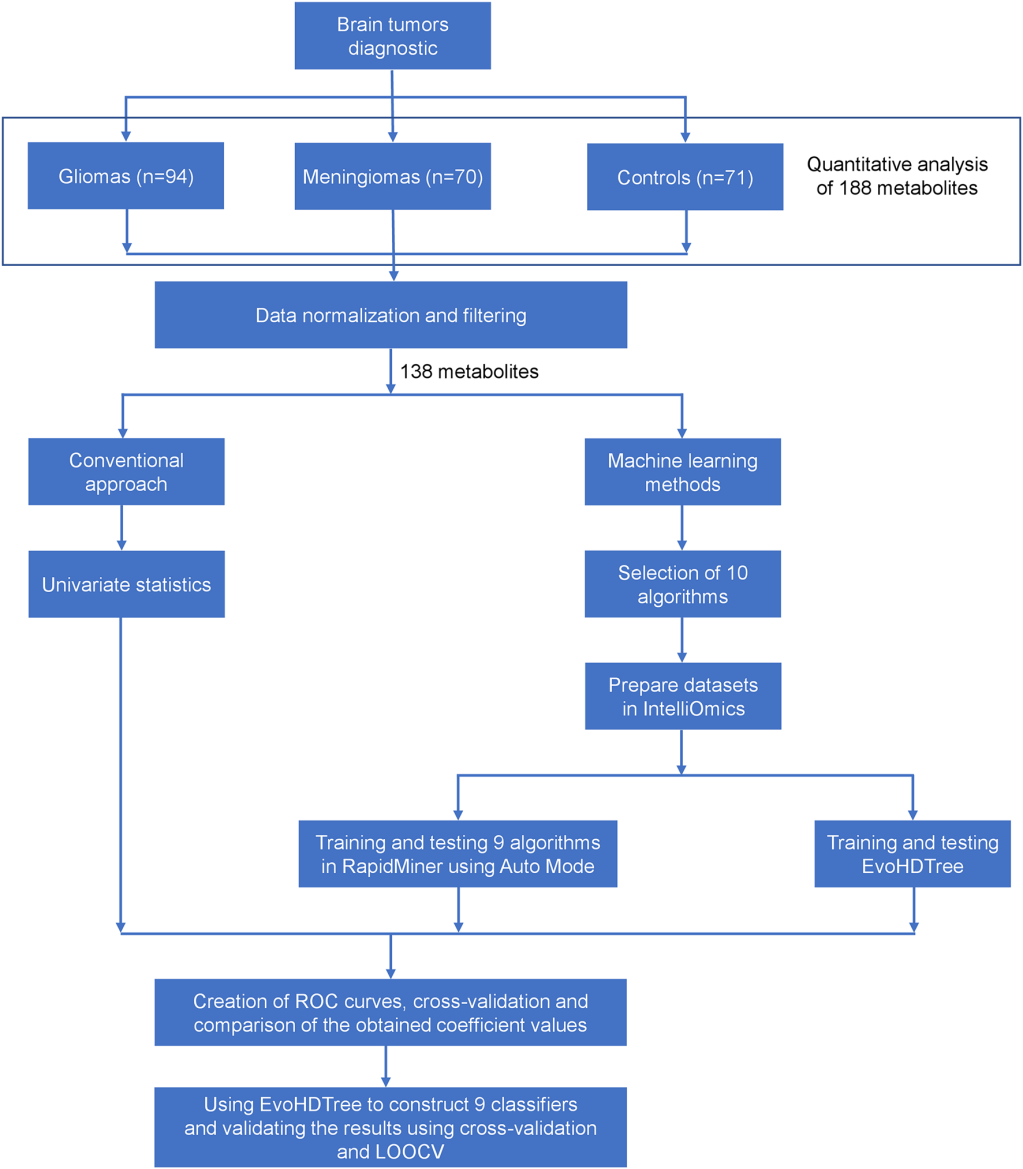
Diagram showing the workflow of the experiment.

Raw spectral data processing, quantification, and normalization were performed using MetIDQ software (Oxygen DB110-3005, Biocrates, Life Science AG, Innsbruck, Austria). Data normalization was performed according to the Biocrates’ kit user manual. The obtained data was combined and filtered accepting only metabolites present in at least 80% of the samples. In such a data matrix, missing values were substituted with half of the limit of detection value for each specific metabolite in each batch. Subsequently, the obtained data matrix containing 138 metabolites was forwarded for MLMs analysis and conventional statistical approach. A diagram showing the workflow is presented in Fig. 5.

### Conventional statistical approach

UVS (the Wilcoxon test or the t-test, depending on the data distribution) was performed using the online tool MetaboAnalyst 5.0. The loaded data was not scaled or transformed. Based on statistically significant metabolites, receiver operating characteristic (ROC) curves with SVM as a classification method were prepared to evaluate the ability of these metabolites to classify study groups.

### Machine learning methods

Ten classification algorithms were used to prepare binary classifiers, i.e.: NB, GLM, LR, FLM, DL, DT, RF, GBT, SVM, and EvoHDTree. With the exception of the last method, all of the aforementioned algorithms are state-of-the-art MLMs. The IntelliOmics platform^71^, was used to prepare and transform the datasets used in the performed experiments. Next, the algorithms were optimized and tested using RapidMiner software, which is one of the most popular and well-established tool in data mining. In the RapidMiner platform, we leveraged the Auto Model module, that, in general, incorporates smart preprocessing steps, which often include handling missing values, outlier treatment, and scaling or transformation, as appropriate for each ML algorithm^72^. EvoHDTree is a new hybrid algorithm in the field of eXplainable Artificial Intelligence, which has until now been used for gene expression data^25^. It combines the power of evolutionary induced DT with a concept called Relative eXpression Analysis (RXA). Notably, the patterns discovered by EvoHDTree, such as DT and LR, are easy to analyze and interpret.

Each algorithm has its own set of specific parameters that can be tuned to improve the performance of the model. Here are some examples of the specific parameters tested for a few commonly used algorithms:

- DT: maximum tree depth and the minimum improvement in splitting;
- RF: number of trees and maximum tree depth;
- SVM: regularization parameter C and hyperparameter Gamma;
- GBT: number of trees, maximum tree depth, learning rate;
- FLM: regularization parameter C;
- DL: uses the adaptive learning rate option;
- EvoHDTree: regularization parameter in the fitness function^25^.

For each algorithm, an automatic search for the best combination of parameter values was used by iterating over a range of possible values and testing each combination against a performance metric (such as accuracy or AUC) to see which produces the best results. The setup and fine-tuning of the parameters were carried out on a subset of the training dataset and performed using Auto Model^72^.

The LOOCV, a standard technique when the number of samples is relatively low, was used for validation, which was performed on data not pre-divided into training and testing parts. This technique reduces overfitting by training the model on all but one of the data points and then validating the model on the left-out data points. The process of classification was carried out without performing any feature selection beforehand, meaning that all available features or variables in the dataset were used in the model. Presented results show an average score of 100 runs due to the existence of nondeterministic algorithms. Along with the confusion matrix, an area under the curve (AUC) and ROC curve were generated for each solution.

## Supplementary Information

Supplementary Table S1.

Supplementary Table S2.

## Data Availability

The data supporting the findings of this study are available as part of the work and are included in the Supplementary Information.

## Abbreviations

AA: Amino acid
ACC: Accuracy
ADMA: Asymmetric dimethylarginine
AUC: Area under the curve
BMI: Body mass index
CNS: Central nervous system
Con: Healthy control
DL: Deep Learning
DT: Decision Tree
EvoHDTree: Evolutionary Heterogeneous Decision Tree
FLM: Fast Large Margin
GI–IV: I–IV grade glioma
GBT: Gradient Boosted Trees
GLM: Generalized Linear Model
HGG: High-grade gliomas
ISTD: Internal standards
LC–MS/MS: Liquid chromatography coupled with tandem mass spectrometry
LGG: Low-grade gliomas
LOOCV: Leave-One-Out Cross-Validation
LR: Logistic Regression
MLM: Machine learning method
MT: Meningioma
NB: Naive Bayes
PC: Phosphatidylcholine
RF: Random forest
ROC: Receiver operating characteristic
RXA: Relative eXpression Analysis
SD: Standard deviation
SDMA: Symmetric dimethylarginine
SM: Sphingomyelin
SVM: Support vector machine
UVS: Univariate statistics
WHO: World Health Organization
